# Prediction of advanced fibrosis in non-alcoholic fatty liver disease using gut microbiota-based approaches compared with simple non-invasive tools

**DOI:** 10.1038/s41598-020-66241-0

**Published:** 2020-06-10

**Authors:** Sonja Lang, Fedja Farowski, Anna Martin, Hilmar Wisplinghoff, Maria J. G. T. Vehreschild, Marcin Krawczyk, Angela Nowag, Anne Kretzschmar, Claus Scholz, Philipp Kasper, Christoph Roderburg, Frank Lammert, Tobias Goeser, Hans-Michael Steffen, Münevver Demir

**Affiliations:** 1 University of Cologne, Faculty of Medicine, and University Hospital Cologne, Department of Gastroenterology and Hepatology, Cologne, Germany; 2 Department of Medicine, University of California San Diego, La Jolla, CA USA; 3 University of Cologne, Department I of Internal Medicine, Center for Integrated Oncology Aachen Bonn Cologne Duesseldorf, Cologne, Germany; 4 German Centre for Infection Research (DZIF), partner site Bonn/Cologne, Cologne, Germany; 5 Department of Internal Medicine, Infectious Diseases, Goethe University Frankfurt, Frankfurt am Main, Germany; 6 Wisplinghoff Laboratories, Cologne, Germany; 7 Institute for Virology and Medical Microbiology, University Witten/Herdecke, Witten, Germany; 8 University of Cologne, Faculty of Medicine, Institute for Medical Microbiology, Immunology and Hygiene, University Hospital of Cologne, Cologne, Germany; 9 Department of Medicine II, Saarland University Medical Center, Homburg, Germany; 10 Laboratory of Metabolic Liver Diseases, Department of General, Transplant and Liver Surgery, Medical University of Warsaw, Warsaw, Poland; 11 Department of Hepatology and Gastroenterology, Campus Virchow Clinic, Charité University Medicine, Berlin, Germany

**Keywords:** Non-alcoholic fatty liver disease, Non-alcoholic steatohepatitis

## Abstract

Liver fibrosis is the major determinant of liver related complications in patients with non-alcoholic fatty liver disease (NAFLD). A gut microbiota signature has been explored to predict advanced fibrosis in NAFLD patients. The aim of this study was to validate and compare the diagnostic performance of gut microbiota-based approaches to simple non-invasive tools for the prediction of advanced fibrosis in NAFLD. 16S rRNA gene sequencing was performed in a cohort of 83 biopsy-proven NAFLD patients and 13 patients with non-invasively diagnosed NAFLD-cirrhosis. Random Forest models based on clinical data and sequencing results were compared with transient elastography, the NAFLD fibrosis score (NFS) and FIB-4 index. A Random Forest model containing clinical features and bacterial taxa achieved an area under the curve (AUC) of 0.87 which was only marginally superior to a model without microbiota features (AUC 0.85). The model that aimed to validate a published algorithm achieved an AUC of 0.71. AUC’s for NFS and FIB-4 index were 0.86 and 0.85. Transient elastography performed best with an AUC of 0.93. Gut microbiota signatures might help to predict advanced fibrosis in NAFLD. However, transient elastography achieved the best diagnostic performance for the detection of NAFLD patients at risk for disease progression.

## Introduction

Non-alcoholic fatty liver disease (NAFLD) is a global health issue with growing incidence^1^. Some patients with simple steatosis develop liver fibrosis which can lead to cirrhosis, hepatocellular carcinoma, liver transplantation and death^2,3^. The presence of liver fibrosis is the major determinant of future liver related complications^4,5^. Liver biopsy, an invasive procedure with an inherent risk of complications remains the gold-standard for disease grading and staging.

There is an urgent need for reliable non-invasive tools to detect NAFLD patients at risk for disease progression in order to prevent further complications. Several non-invasive tests such as the NAFLD fibrosis score (NFS)^6^ and FIB-4 index^7^ have been developed and included into a diagnostic algorithm proposed by the European Association for the Study of the Liver^8^.

Recent studies have further linked alterations in the gut microbiota to disease severity in NAFLD^9–19^. Loomba et al. were the first, who precisely and non-invasively identified NAFLD patients with advanced fibrosis (F3-F4) by using a machine learning algorithm which combined the abundance of specific gut microbial taxa and clinical features^15^. However, this Random Forest algorithm has not been validated so far.

The aim of this study was to validate the diagnostic accuracy of the gut microbiota-based prediction model published by Loomba *et al*.^15^, to compare the results to an own Random Forest model including clinical features and 16S rRNA gene sequencing data and to compare the results to the NFS and FIB-4 index as well as transient elastography as ultrasound-based method for the prediction of advanced fibrosis (fibrosis stage F3-F4) in 96 NAFLD patients.

## Methods

### Patient cohort

This cross-sectional, prospective study was primarily designed to detect associations between the gut microbiota, diet, as well as genetic factors with the activity and severity of NAFLD. Therefore, a total of 180 NAFLD patients were prospectively enrolled between March 2015 and December 2018 in the outpatient liver department of the Clinic for Gastroenterology and Hepatology, University Hospital of Cologne, Germany. The protocol was approved by the Ethics Commission of Cologne University’s Faculty of Medicine and written informed consent was obtained from each patient. The study was performed in accordance with the Declaration of Helsinki.

In this secondary analysis, 83 biopsy-proven NAFLD patients and 13 NAFLD patients diagnosed with cirrhosis based on characteristic clinical findings (see criteria below) were included.

Patients were referred to our tertiary referral center with elevated liver function tests and/or liver abnormalities on ultrasound for further diagnostic tests, or with already diagnosed NAFLD in order to investigate disease activity and severity. If NAFLD diagnosis was made or confirmed, patients were consecutively enrolled in this observational study, after written informed consent was obtained.

Within the study, a detailed history, physical exam, anthropometric measurements, blood pressure measurements, ultrasound and/or magnetic resonance imaging (MRI), transient elastography and liver biopsy, if clinically indicated, as per standard of care were performed. NAFLD was diagnosed, if the following conditions were met: hepatic steatosis on liver imaging (ultrasound and/or magnet resonance imaging) and/or the presence of >5% fat in histological analysis of liver biopsy, daily alcohol consumption of less than 10 g in women and less than 20 g in men, absence of steatogenic drugs such as glucocorticoids, methotrexate, amiodarone and tamoxifen, absence of other diseases causing secondary steatosis such as human immunodeficiency virus infection, celiac disease or inflammatory bowel disease, absence of other chronic liver diseases, e.g. viral hepatitis, autoimmune hepatitis, toxic liver injury, alcoholic steatohepatitis, cholestatic liver disease, Wilson’s disease and hereditary hemochromatosis. Alcohol consumption was self-reported. The daily alcohol intake was calculated based on the regular amount of specific alcoholic beverages according to patients’ disclosures. Exclusion criteria for all study subjects were oral- or intravenous antibiotic treatment within the last 6 months prior to the study, known malignancy, pregnancy, and age <18 years. Any recommendations or treatment suggestions for study participants did not differ from usual patient care. Thus, NAFLD patients received the same overall lifestyle recommendations as indicated in the current European guideline^20^. Further exclusion criteria for NAFLD patients were ongoing successful lifestyle modifications defined as more than 5% loss of body weight within the last three months prior to enrollment or current or prior participation in an interventional non-alcoholic steatohepatitis (NASH) study^21^.

Abdominal ultrasound was performed for all patients. All blood samples for laboratory analyses were collected under fasting conditions. Anthropometric measurements were carried out by trained physicians or research assistant nurses.

Type 2 diabetes was defined as glycated hemoglobin (HbA1c) ≥6.5% and/or fasting glucose ≥126 mg/dL and/or use of antidiabetic medications. Overweight was defined as body mass index (BMI) ≥ 25 kg/m^2^. Metabolic syndrome was defined following the International Diabetes Foundation (IDF) criteria. Arterial hypertension was defined as office blood pressure ≥140/90 mmHg on ≥2 measurements during ≥2 occasions or antihypertensive drug treatment.

### Liver biopsies

Liver biopsy was performed in patients with elevated liver function test values over a longer period, to rule out potential other liver diseases than NAFLD and if a possible underlying advanced liver injury was suggested. Indicators for an advanced liver injury included altered laboratory parameters, co-morbidities, findings from ultrasound and/or MRI and results from transient elastography measurements.

If liver biopsy was performed, samples were evaluated by an experienced liver pathologist who was blinded for all clinical and laboratory patient data. The NASH clinical research network histological scoring system^22^ was used to evaluate disease activity and severity. Accordingly, steatosis was graded 0–3, ballooning was graded 0–2, lobular inflammation was graded 0–3. Fibrosis was staged from 0–4. Stages 1a, 1b and 1c were summarized as stage 1. Fibrosis stages: 0 none, 1 perisinusoidal or periportal, 2 perisinusoidal and portal/periportal, 3 bridging fibrosis, 4 cirrhosis. The NAFLD activity score was obtained for each biopsy. This score is defined as the unweighted sum of the scores for steatosis, (0–3), lobular inflammation (0–3), and ballooning (0–2); thus ranging from 0 to 8^21,22^.

### Non-invasive diagnosis of liver cirrhosis

If the following criteria were present, patients were staged as histological F4 fibrosis without determination of histological grading: liver imaging consistent with liver cirrhosis (e.g. nodular hepatic contour, changes in volume distribution indicating portal hypertension in the absence of portal vein thrombosis, secondary phenomena of portal hypertension such as splenomegaly, enlarged caudate lobe and left lobe lateral segment, regenerative nodules) together with clinical and laboratory signs of portal hypertension/cirrhosis (e.g. low platelets, albumin and prothrombin time, esophageal varices)^21,23^.

### Liver stiffness measurement

For all patients, vibration controlled transient elastography (FibroScan, Echosens, Paris, France) was performed in fasting patients by experienced operators, blinded to all clinical patient data. At least 10 valid measurements were performed, and the median value of these measurements was reported in kPa. In accordance with the manufacturer’s protocol, patients were first scanned using the M probe and if indicated by the equipment, patients were re-scanned using the XL probe. Sensitivity, specificity, positive-, and negative predictive values were calculated using published cut-off values^24^.

### Non-invasive fibrosis tests

The fibrosis-4 index (FIB-4)^7^ and NAFLD fibrosis score^6^ were calculated for each patient. Sensitivity, specificity, positive-, and negative predictive values were calculated using published cut-off values^8^.

### Gut bacterial sequencing

The DNA was isolated using the RNeasy Power Microbiome Kit (Qiagen, Hilden, Germany). Seven of the 9 variable bacterial 16S rRNA gene regions (pool 1: V2, V4 and V8; pool 2: V3, V6/7 and V9) were amplified with the Ion 16S Metagenomics Kit (Thermo Fisher Scienctific, Waltham, USA) utilizing two primer pools (An integrated research solution for bacterial identification using 16S rRNA sequencing on the Ion PGM System with Ion Reporter Software https://www.thermofisher.com/content/ dam/LifeTech/Documents/PDFs/Ion-16S-Metagenomics-Kit-Software-Application-ote.pdf). Amplicons were pooled and cleaned using the NucleoMag NGS Clean-up (Macherey-Nagel, Düren, Germany). The Qubit system was used to determine amplicon concentration, the library was prepared with the Ion Plus Fragment Library Kit (Thermo Fisher Scienctific, Waltham, USA). For the template-preparation amplicon concentration was diluted to 30 ng/mL. The Ion Chef Kit and the Ion Chef system (both, Thermo Fisher Scienctific, Waltham, USA) were used to enrich and prepare the template-positive Ion Sphere Particles (ISP). Amplicon library was sequenced using the Ion Torrent S5 system (pH-dependent, Thermo Fisher Scienctific, Waltham, USA). The amplicon sequences were clustered into operational taxonomic units (OTUs) before taxonomical alignment with the MicroSEQ. 16S-rDNA Reference Library v2013.1 (Thermo Fisher Scienctific, Waltham, USA) and Greengenes v13.5 databases. 97% similarity was used to genus level assignment and 99% similarity for species level assignment. Data files were assigned by the Ion Reporter metagenomics 16S w1.1 workflow (Thermo Fisher Scienctific, Waltham, USA). The raw data was processed using the programming language R version 3.5.1.

### Accession numbers sequence data

Sequence data were registered at NCBI under BioProject PRJNA540738. Sequence reads are available at NCBI under the following BioSample IDs: SAMN11554417-SAMN11554433, SAMN11554446, SAMN11554451-SAMN11554484 and SAMN13895357- SAMN13895400.

### Statistical analysis

Results are expressed as median and interquartile range in parentheses for each continuous outcome and as number and percentage for categorical variables. A two-sided *P* value of equal or less than 0.05 was considered as statistically significant. Comparisons of clinical characteristics between groups were performed using the Kruskal-Wallis test with Dunn’s post-hoc test for continuous and Fisher’s exact test for categorical variables, each followed by false discovery rate (FDR) procedures to correct for multiple comparisons.

According to Loomba *et al*.^15^ we used a Random Forest machine learning algorithm to identify taxa and clinical variables that predict advanced fibrosis. In order to reduce noise, only taxa present in at least 66% of all samples and with a mean relative abundance >10^−4^ were considered as an input feature. Features resulting in the “best” accuracy were selected by the recursive feature elimination algorithm. The dataset was repeatedly (300 times) randomly split into training and test datasets. We further trained a Random Forest model including only overlapping taxa and the same clinical variables as implemented by Loomba *et al*.^15^. Only 16 species out of 37 species identified by Loomba et al. were also detected in our cohort. For these unresolved species, we included all other species within the respective genus. E.g. for “*Blautia* sp. KLE 1732” and “*Blautia* sp. CAG:37”, we included *Blautia coccoides, Blautia faecis, Blautia glucerasea, Blautia hansenii, Blautia hydrogenotrophica, Blautia luti, Blautia obeum, Blautia producta, Blautia stercoris, Blautia wexlerae, Ruminococcus gnavus, Ruminococcus torques and Blautia* unknown species, which resulted in 136 taxa. To increase the diagnostic accuracy and to reduce distraction of the model, we used Random Forest feature elimination to determine the top 37 taxa out of these 136 features together with age, Shannon diversity index, gender and BMI.

Receiver operating characteristic (ROC) analysis with calculation of the area under the curve (AUC) was performed to compare all non-invasive approaches. For the clinical scores and transient elastography, we calculated sensitivity, specificity, positive-, and negative predictive values using published cut-offs^8,24^. Statistical analysis was performed using R statistical software, R version 3.5.1, 2018 the R Foundation for Statistical Computing. This report follows the Standards for Reporting Diagnostic accuracy studies (STARD) checklist^25^.

## Results

A total of 65 patients was staged as none to significant (F0-F2) fibrosis and compared with 31 patients with advanced (F3-F4) fibrosis of whom 13 patients were staged as F4 fibrosis based on characteristic clinical findings (see Methods sections). Patients with advanced fibrosis were older, had a higher BMI and waist-circumference, suffered more frequently from type 2 diabetes, arterial hypertension and metabolic syndrome, differed significantly in a variety of laboratory parameters and used proton pump inhibitors more frequently on a daily basis (Table 1). Patients with cirrhosis diagnosed based on characteristic clinical findings had significantly higher bilirubin (*P* = 0.006) and international normalized ratio (INR) (*P* = 0.04) levels, lower platelet counts (*P* = 0.003), a higher NAFLD fibrosis score (*P* = 0.023) and FIB-4 Index (*P* = 0.028) compared to those patients with advanced fibrosis detected at liver biopsy (Supplementary Table 1).

**Table 1:** Characteristics of the study cohort.

	N/A	Biopsy-proven F0-F2	Biopsy-proven F3-F4	Non-invasive F4	*P* value
Total n		65	18	13	
**Demographics**					
Age, years		50.0 (23.0)	59.0 (14.0)	64.0 (7.0)	**<0.001**
Gender female, n (%)		30 (46.2)	9 (52.9)	5 (38.5)	0.739
Body mass index, kg/m²		30.0 (6.2)	32.1 (10.2)	31.4 (4.3)	0.053
Type 2 diabetes, n (%)		7 (10.8)	9 (52.9)	7 (53.8)	**<0.001**
Arterial hypertension, n (%)		37 (56.9)	14 (82.4)	11 (84.6)	**0.042**
Metabolic syndrome (IDF criteria), n (%)	1	20 (31.2)	11 (64.7)	8 (61.5)	**0.012**
Waist circumference (cm)	18	105.5 (19.8)	119.5 (19.2)	120.0 (4.2)	**0.003**
Metformin use, n (%)		7 (10.8)	5 (29.4)	5 (38.5)	**0.024**
Antihypertensive drug use, n (%)		26 (40.0)	12 (70.6)	10 (76.9)	**0.010**
Proton pump inhibitor use, n (%)		5 (7.7)	5 (29.4)	4 (30.8)	**0.018**
**Laboratory parameters**					
Albumin, g/L	1	45.0 (3.5)	44.0 (4.0)	42.0 (3.0)	**0.025**
Creatinine, mg/dL	1	0.8 (0.3)	0.8 (0.2)	0.9 (0.2)	0.403
Urea, mg/dL	1	29.0 (14.0)	26.0 (11.0)	29.0 (12.0)	0.557
Uric acid, mg/dL	1	6.1 (2.0)	6.1 (1.9)	6.2 (2.4)	0.666
AST, U/L	1	32.5 (21.0)	51.0 (26.0)	56.0 (23.0)	**0.001**
ALT, U/L	1	50.5 (53.5)	59.0 (28.0)	34.0 (34.0)	0.122
GGT, U/L	1	67.0 (82.5)	119.0 (79.0)	180.0 (103.0)	**0.002**
Alkaline phosphatase, U/L	1	73.5 (26.0)	81.0 (27.0)	90.0 (37.0)	0.195
Bilirubin, mg/dL	2	0.5 (0.4)	0.4 (0.3)	0.9 (0.5)	**0.005**
Ferritin, µg/L	2	180.0 (168.0)	238.0 (165.0)	195.5 (278.0)	0.439
Triglycerides, mg/dL	1	137.0 (105.8)	197.0 (130.0)	129.0 (142.0)	0.162
Total cholesterol, mg/dL	1	189.0 (56.2)	171.0 (57.0)	169.0 (42.0)	0.055
HDL cholesterol mg/dL	6	50.0 (20.0)	43.0 (10.5)	41.0 (14.8)	0.092
LDL cholesterol mg/dL	9	119.5 (48.8)	99.0 (63.0)	81.0 (56.5)	0.058
Platelet count, x1E9/L	1	225.0 (85.5)	234.0 (90.0)	121.0 (70.0)	**<0.001**
INR	1	1.0 (0.1)	1.0 (0.1)	1.1 (0.3)	**<0.001**
Prothrombin time (s)	1	108.0 (16.2)	105.0 (25.0)	84.0 (41.0)	**<0.001**
HbA1c, %	10	5.2 (0.5)	5.9 (1.2)	6.0 (1.1)	**<0.001**
Fasting glucose, mg/dL	1	93.5 (14.0)	110.0 (37.0)	131.0 (56.0)	**<0.001**
Alpha-fetoprotein kU/L	11	2.0 (2.0)	4.0 (2.0)	4.0 (0.0)	**<0.001**
**Non-invasive fibrosis assessment**					
Transient Elastography, kPa	5	5.4 (2.4)	13.6 (6.6)	24.4 (16.0)	**<0.001**
NAFLD Fibrosis Score	1	−2.5 (2.1)	−0.7 (1.3)	0.9 (1.3)	**<0.001**
FIB-4 Index	1	0.9 (0.8)	1.8 (1.4)	3.8 (3.1)	**<0.001**

Values are presented as median and interquartile range (IQR) in brackets. 65 NAFLD patients were staged as F0-F2 fibrosis based on their liver biopsy result (“biopsy-proven F0-F2”), 18 NAFLD patients were staged as F3-F4 fibrosis based on their liver biopsy result (“biopsy-proven F3-F4”) and 13 patients were staged as NAFLD-cirrhosis based on characteristic findings on ultrasound and/or magnetic resonance imaging together with clinical and laboratory findings (“non-invasive F4”) (see Methods section for details). Groups were compared using the Kruskal-Wallis test with Dunn’s post-hoc test for continuous and Fisher’s exact test for categorical variables, each followed by false discovery rate (FDR) procedures to correct for multiple comparisons. Bold font indicates significance (*P* value < 0.05). Post-hoc *P* values for significant variables are reported in Supplementary Table 1. The number of missing values within the overall cohort is indicated in the third column (“N/A”). ALT, alanine aminotransferase; AST, aspartate aminotransferase; BMI, body mass index; GGT, gamma-glutamyl-transferase; HbA1c, glycated hemoglobin; INR, international normalized ratio; HDL, High-density lipoprotein; kPa, kilopascal; LDL, low-density lipoprotein.

All liver histology features are shown in Table 2.

**Table 2:** Liver histology features of the cohort.

Liver histology feature	Scoring	Classification	NAFLD F0–F2	NAFLD F3–F4
Total n			65	18
Grade of steatosis, n (%)	0	<5%	0	0
	1	5%–33%	20 (30.8)	5 (27.8)
	2	>33%–66%	29 (44.6)	6 (33.3)
	3	>66%	16 (24.6)	7 (38.9)
Ballooning, n (%)	0	none	18 (27.7)	1 (5.6)
	1	few balloon cells	33 (50.8)	6 (33.3)
	2	prominent ballooning	14 (21.5)	11 (61.1)
Grade of inflammation, n (%)	0	no foci	10 (15.4)	1 (5.6)
	1	<2 foci	39 (60.0)	5 (27.8)
	2	2–4 foci	16 (24.6)	10 (55.6)
	3	>4 foci	0	2 (11.1)
Fibrosis stage, n (%)	0	None	20 (30.8)	
	1	Perisinusoidal or periportal	29 (44.6)	
	2	Perisinusoidal and portal/periportal	16 (24.6)	
	3	Bridging fibrosis		8 (44.4)
	4	Cirrhosis		10 (55.6)

Liver histology features of 83 NAFLD patients who underwent liver biopsy.

### Random Forest models to predict advanced fibrosis

**Figure 1:**
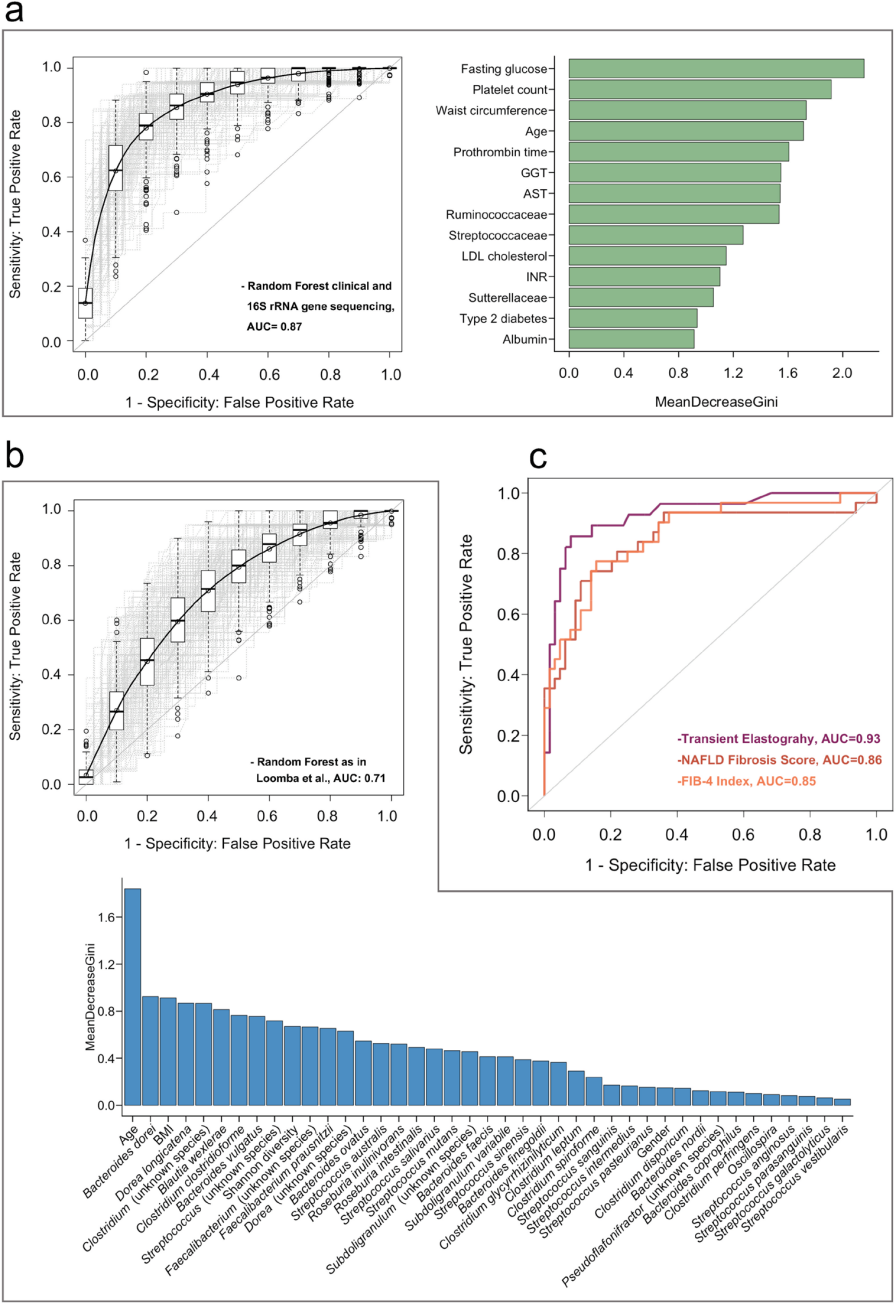
Comparison of Random Forest models with simple non-invasive tools to predict advanced fibrosis in NAFLD. (**a**) Area under the curve (AUC) for our Random Forest model based on 14 features (right panel) that were identified by Random Forest feature elimination. Light grey lines represent the 300 training runs, the black line and AUC represent the median over these. The right panel shows the feature importance based on mean decrease in Gini index. All shown bacterial taxa belong to the family level (**b**) AUC and mean decrease in Gini index for the validation approximation of the Random Forest model by Loomba *et al*. Only 16 species out of 37 species identified by Loomba *et al*. were also detected in our cohort. For these unresolved species, we included all other species within the respective genus (see methods section) which resulted in 136 taxa. To increase the diagnostic accuracy, we used Random Forest feature elimination to determine the top 37 taxa out of these 136 features together with age, Shannon diversity, gender and BMI. (**c**) Diagnostic performance of the FIB-4 index, NAFLD fibrosis score and transient elastography. In a-c, 83 biopsy-proven NAFLD patients and 13 NAFLD patients diagnosed with liver cirrhosis based on clinical characteristic and characteristic findings on liver imaging (see criteria in methods section) were included. 65 patients were staged as F0-F2 and 31 as F3-F4. AST, aspartate aminotransferase; GGT, gamma-glutamyl-transferase; INR, international normalized ratio; LDL, low-density lipoprotein; FIB-4, fibrosis-4 index.

Our Random Forest model, including all laboratory parameters from Table 1 together with age, gender, BMI, type 2 diabetes, arterial hypertension, metabolic syndrome, waist circumference and all gut bacterial taxa that met the criteria as an input feature, identified Ruminococcaceae (family), Streptococaceae (family) and Sutterellaceae (family) as gut bacterial predictors of advanced fibrosis in NAFLD. The strongest predictors, however, were clinical features such as fasting glucose, platelet count and age. This Random Forest model, based on clinical variables and the mentioned gut microbial taxa, achieved an AUC of 0.87 (95% CI 0.865–0.874, Fig. 1a).

We further compared the data to a model limited to clinical variables. In this model, fasting glucose, platelet counts, waist circumference, age, gamma-glutamyl-transferase (GGT), prothrombin time and aspartate aminotransferase (AST), followed by INR, albumin and type 2 diabetes were predictors of advanced fibrosis, identified by Random Forest feature elimination. The resulting AUC achieved 0.85 (95% CI 0.849–0.858, Supplementary Fig. 1a), indicating that the gut microbiota component of the previous model did not add major value regarding prediction of advanced fibrosis compared with the model which only included clinical variables.

Sixteen gut bacterial taxa were overlapping with the Random Forest model published by Loomba *et al*.^15^. To cover the remaining specific 21 species, we included all other related species within the corresponding genus level for the respective undetected species (see methods section). In this dataset, we used Random Forest feature elimination to select only the top 37 taxa together with Shannon diversity index, age, gender and BMI (according to Loomba *et al*.^15^). This model achieved an AUC of 0.71 (95% CI 0.701–0.714) for the prediction of advanced fibrosis in NAFLD (Fig. 1b).

### Comparison of random forest models to conventional non-invasive fibrosis tests

We further compared gut microbiota-based approaches to well-known simple non-invasive fibrosis scores based on clinical and laboratory parameters as well as transient elastography in our cohort. The NAFLD fibrosis score and the FIB-4 index achieved both comparable results with an AUC of 0.86 (95% CI 0.75–0.94) and 0.85 (95% CI 0.78–0.94), respectively (Fig. 1c).

Transient elastography had the highest diagnostic performance with an AUC of 0.93 (95% CI 0.87–0.99, Fig. 1c). We used published cut-off values to further determine the diagnostic sensitivity, specificity, positive and negative predictive values of the NFS and FIB-4 (Table 3). When we compared the number of patients classified into intermediate stages to the actual, by liver biopsy confirmed fibrosis stage, both non-invasive fibrosis scores classified a considerable number of patients into intermediate stages. In contrast, this was only observed for two patients when using transient elastography. Transient elastography had the highest negative predictive value (94.6%) whereas the highest positive predictive value was observed for the FIB-4 index (85.7%, Table 3).

**Table 3:** Diagnostic performance of non-invasive fibrosis tests.

Transient Elastography	<7.9	7.9–9.6	>9.6
F0-2 (n)	53	1	9
F3-4 (n)	3	1	24
Sensitivity (%)	89.3		85.7
Specificity (%)	84.1		85.7
Negative predictive value (%)	94.6		93.1
Positive predictive value (%)	71.4		72.7
FIB-4 Index	<1.30	1.30–3.25	>3.25
F0-2 (n)	46	16	2
F3-4 (n)	5	14	12
Sensitivity (%)	83.9		38.7
Specificity (%)	71.9		96.9
Negative predictive value (%)	90.2		76.5
Positive predictive value (%)	59.1		85.7
NAFLD Fibrosis Score	<−1.455	−1.455–0.676	>0.676
F0-2 (n)	46	17	1
F3-4 (n)	6	13	12
Sensitivity (%)	80.6		71.0
Specificity (%)	71.9		85.9
Negative predictive value (%)	88.5		85.9
Positive predictive value (%)	58.1		71.0

83 biopsy-proven NAFLD patients and 13 NAFLD patients diagnosed with liver cirrhosis based on characteristic clinical findings (see criteria in methods section) were included. Due to present missing values, 91 patients were included in the analysis for transient elastography, 95 for the FIB-4 analysis and 95 for the analysis NAFLD fibrosis score analysis.

Overall, the prediction of advanced fibrosis with transient elastography as an imaging-based method, performed superior when compared with Random Forest classifier models based on the gut bacterial microbiota together with clinical data or clinical features alone.

## Discussion

In this study, we directly compared the performance of widely used non-invasive tools to microbiota-based machine learning approaches for the detection of advanced fibrosis in a well-described cohort of biopsy-proven NAFLD patients. Transient elastography, which is a fast and convenient method performed best, with an AUC of 0.93 for the prediction of advanced fibrosis.

Alterations in the gut microbiota have been linked to NAFLD but no consistent disease-specific gut microbiota signature has been established across several studies including patients with different geographical, ethnic and dietary backgrounds^9–19^. Further, using different sequencing methods limits the comparability of individual studies. While most studies, including our own, used 16S rRNA gene sequencing methods, the study by Loomba et al. sequenced the complete metagenome of gut microbial communities. The model by Loomba et al. includes bacterial taxa at species level and several of the included species could not be resolved in our study cohort. The analysis of 16s rRNA gene sequencing data involves clustering of the obtained sequences into OTUs. This approach is well-established, widely used and efficient. However, several challenges remain in terms of accurate and precise taxonomic quantification at species level^26^. On the other hand, assembling genomes from whole genome sequencing can be more informative and precise in identifying species level but may fail to identify the taxonomic origins of a gene of interest or to produce accurate and unbiased estimates of gene families abundances^26^.

Besides differences in the technical approaches, studied patient populations differed in several aspects. NAFLD patients in the study by Loomba et al. were enrolled in the Southwestern United States and included 34% NAFLD patients with Hispanic ethnicity while our study, performed in Germany, includes almost exclusively patients with a white ethnic background. Region-specific variations in lifestyle, ethnicity, nutrition, medication, genetics and environmental conditions play a role in shaping the gut microbiome. While some bacterial taxa are found to be common in all populations from different countries, abundances of multiple taxa have been found to vary substantially across populations^27^.

These aspects altogether might explain why we did not identify the same gut bacterial taxa at species level in our patient cohort and why several identified taxa by Loomba et al. were not similarly associated with advanced fibrosis in our study cohort. For a conclusive and more accurate validation, the combination of both datasets within one Random Forest algorithm using the same NGS method seems to be essential to distinguish between differences in the gut microbiome composition due to different NGS methods versus variations in patient populations.

Using an own Random Forest algorithm, we were able to achieve a good diagnostic accuracy. This algorithm was, however, still inferior to transient elastography. Compared with a Random Forest model consisting only of clinical features, adding bacterial taxa did not add major value to the model performance. This indicates that clinical features might be still more consistently associated with progression of liver disease compared with the relatively inconsistent findings from studies investigating gut microbiota alterations in NAFLD patients.

In conclusion, among the tested modalities to non-invasively predict advanced fibrosis in NAFLD patients, transient elastography, which is an easily applicable ultrasound-based method, performed best with an excellent diagnostic performance compared with other simple non-invasive scores and gut microbiota-based approaches in our cohort. When NGS will become easier applicable and standards regarding NGS based methods are more established, assessment of the gut microbiome might help to identify NAFLD patients with ongoing disease progression with the aim to prevent further liver related complications.

## Supplementary information

Supplementary information.
