# Supplementary material for: Prediction of advanced fibrosis in non-alcoholic fatty liver disease using gut microbiota-based approaches compared with simple non-invasive tools
Source: Sci Rep. 2020 Jun 10;10:9385. doi: 10.1038/s41598-020-66241-0 (PMC7286895; doi:10.1038/s41598-020-66241-0)
Supplement: Supplementary file 1 — Supplementary information. [file 41598_2020_66241_MOESM1_ESM.pdf]

SUPPLEMENTARY MATERIAL FOR:

Prediction of advanced fibrosis in non-alcoholic fatty liver disease using gut microbiota-based approaches compared with simple non-invasive tools

Sonja Lang[1,2], Fedja Farowski[3,4,5], Anna Martin[1], Hilmar Wisplinghoff[6,7,8], Maria J.G.T. Vehreschild[3,4,5], Marcin Krawczyk[9,10], Angela Nowag[6,8], Anne Kretzschmar[6], Claus Scholz[6], Philipp Kasper[1], Christoph Roderburg[11], Frank Lammert[9], Tobias Goeser[1], Hans-Michael Steffen[1], Münevver Demir[1,11]

[1]University of Cologne, Faculty of Medicine, and University Hospital Cologne, Department of Gastroenterology and Hepatology, Cologne, Germany, [2]Department of Medicine, University of California San Diego, La Jolla, CA, USA, [3]University of Cologne, Department I of Internal Medicine, Center for Integrated Oncology Aachen Bonn Cologne Duesseldorf, Cologne, Germany, [4]German Centre for Infection Research (DZIF), partner site Bonn/Cologne, [5]Department of Internal Medicine, Infectious Diseases, Goethe University Frankfurt, Frankfurt am Main, Germany, [6]Wisplinghoff Laboratories, Cologne, Germany, [7]Institute for Virology and Medical Microbiology, University Witten/Herdecke, Witten, Germany, [8]University of Cologne, Faculty of Medicine, Institute for Medical Microbiology, Immunology and Hygiene, University Hospital of Cologne, Cologne, Germany, [9]Department of Medicine II, Saarland University Medical Center, Homburg, Germany, [10]Laboratory of Metabolic Liver Diseases, Department of General, Transplant and Liver Surgery, Medical University of Warsaw, Warsaw, Poland, [11]Department of Hepatology and Gastroenterology, Campus Virchow Clinic, Charité University Medicine, Berlin, Germany.

Supplementary Figure 1

a

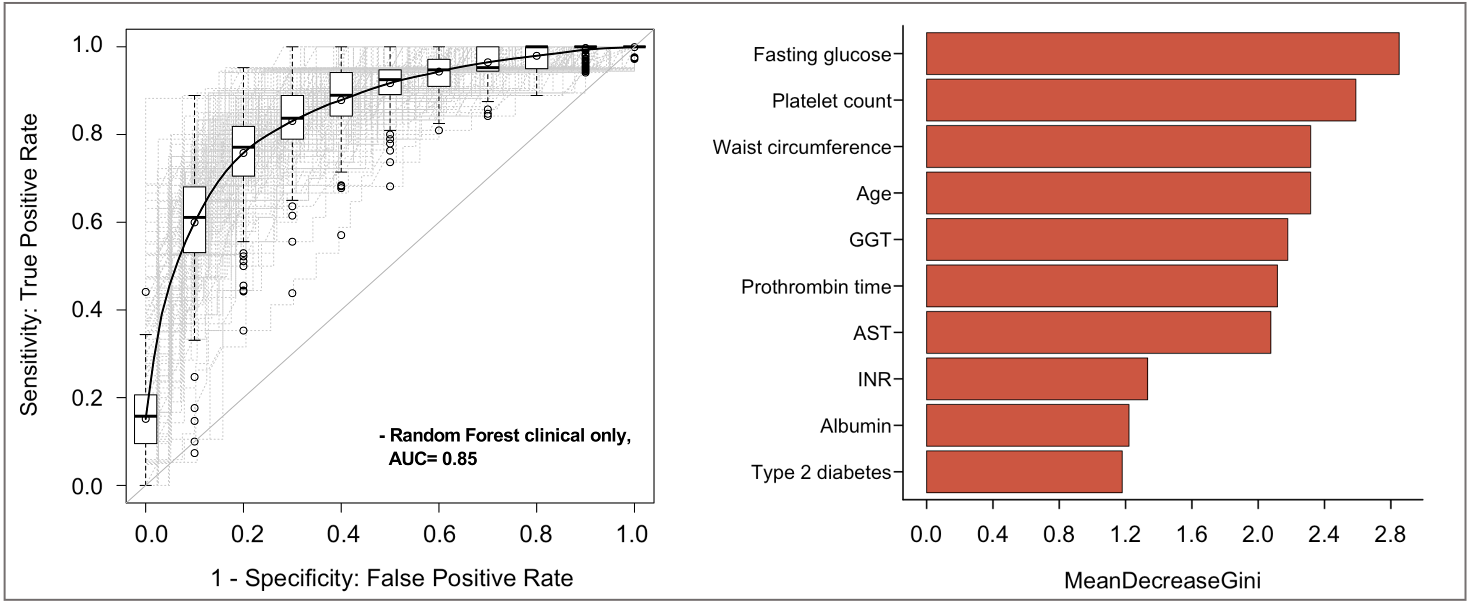

Supplementary Fig. 1. Random Forest model based on clinical features
(a) Area under the curve (AUC) for our Random Forest model based on 10 clinical features (right panel) that were identified by Random Forest feature elimination. The right panel shows the feature importance based on Mean decrease in Gini index. 84 biopsy-proven NAFLD patients and 12 NAFLD patients diagnosed with liver cirrhosis based on characteristic clinical and imaging findings (see criteria in Methods section) were included. 65 patients were staged as F0-F2 and 31 as F3-F4. AST, aspartate aminotransferase; GGT, gamma-glutamyl-transferase; INR, international normalized ratio; LDL, Low-density lipoprotein; FIB-4, fibrosis-4 index.

Supplementary Table 1

	Biopsy-proven F0-F2 vs. F3-F4	Biopsy-proven F0-F2 vs. non-invasive F4	Biopsy-proven F3-F4 vs. non-invasive F4
**Demographics**			
Age	**0.04**	**<0.001**	0.083
Type 2 diabetes	**0.001**	**0.002**	1
Arterial hypertension	0.135	0.135	1
Metabolic syndrome	0.068	0.086	1
Waist circumference	**0.038**	**0.01**	0.313
Metformin use	0.114	0.054	0.524
Antihypertensive drug use	**0.038**	**0.038**	0.732
Proton pump inhibitor use	**0.050**	**0.050**	0.918
**Laboratory parameters**			
Albumin	0.195	**0.037**	0.345
AST	**0.006**	**0.015**	0.869
GGT	**0.047**	**0.005**	0.312
Bilirubin	0.376	**0.006**	**0.006**
Platelet count	0.699	**<0.001**	**0.003**
INR	0.071	**<0.001**	**0.04**
Prothrombin time	0.180	**<0.001**	0.051
HbA1c	**0.001**	**0.004**	0.759
Fasting glucose	**0.042**	**0.001**	0.168
Alpha-fetoprotein	**0.003**	**0.003**	0.742
**Non-invasive fibrosis assessment**			
Transient Elastography	**<0.001**	**<0.001**	0.123
NAFLD Fibrosis Score	**0.002**	**<0.001**	**0.023**
FIB-4 Index	**0.003**	**<0.001**	**0.028**

Post-hoc *P* values for significant variables reported in Table 1. 65 NAFLD patients were staged as F0-F2 fibrosis based on their liver biopsy result (“biopsy-proven F0-F2”), 18 NAFLD patients were staged as F3-F4 fibrosis based on their liver biopsy result (“biopsy-proven F3-F4”) and 13 patients were staged as NAFLD-cirrhosis based on characteristic findings on ultrasound and/or magnetic resonance imaging together with clinical and laboratory findings (“non-invasive F4”) (see Methods section). Groups were compared using the Kruskal-Wallis test with Dunn’s post-hoc test for continuous and Fisher’s exact test for categorical variables, each followed by false discovery rate (FDR) procedures to correct for multiple comparisons. Bold font indicates significance (*P* value <0.05). AST, aspartate aminotransferase; GGT, gamma-glutamyl-transferase; HbA1c, glycated hemoglobin; INR, international normalized ratio.
